# Limitations to estimating bacterial cross-species transmission using genetic and genomic markers: inferences from simulation modeling

**DOI:** 10.1111/eva.12173

**Published:** 2014-07-23

**Authors:** Julio A Benavides, Paul C Cross, Gordon Luikart, Scott Creel

**Affiliations:** 1 Department of Ecology, Montana State UniversityBozeman, MT, USA; 2 U.S. Geological Survey, Northern Rocky Mountain Science CenterBozeman, MT, USA; 3 Flathead Lake Biological Station, Fish and Wildlife Genomics Group, Division of Biological Sciences, University of MontanaPolson, MT, USA

**Keywords:** bacterial pathogens, cross-species transmission, infectious disease, molecular epidemiology, most parsimonious phylogenetic reconstruction, simulation modeling

## Abstract

Cross-species transmission (CST) of bacterial pathogens has major implications for human health, livestock, and wildlife management because it determines whether control actions in one species may have subsequent effects on other potential host species. The study of bacterial transmission has benefitted from methods measuring two types of genetic variation: variable number of tandem repeats (VNTRs) and single nucleotide polymorphisms (SNPs). However, it is unclear whether these data can distinguish between different epidemiological scenarios. We used a simulation model with two host species and known transmission rates (within and between species) to evaluate the utility of these markers for inferring CST. We found that CST estimates are biased for a wide range of parameters when based on VNTRs and a most parsimonious reconstructed phylogeny. However, estimations of CST rates lower than 5% can be achieved with relatively low bias using as low as 250 SNPs. CST estimates are sensitive to several parameters, including the number of mutations accumulated since introduction, stochasticity, the genetic difference of strains introduced, and the sampling effort. Our results suggest that, even with whole-genome sequences, unbiased estimates of CST will be difficult when sampling is limited, mutation rates are low, or for pathogens that were recently introduced.

## Introduction

Bacterial cross-species transmission (CST) is of major concern for public health, agriculture, and wildlife management. First, CST is the most significant cause of disease emergence in humans and other species (Lloyd-Smith et al. 2009), with wildlife zoonotic diseases of bacterial origin being the most common group of human emerging diseases (Jones et al. 2008). Secondly, CST between wildlife and livestock for diseases such as tuberculosis and brucellosis has appreciable economic impacts in agriculture by reducing livestock productivity and imposing export restrictions (Gortázar et al. 2007). As a result, wild and domestic species are sometimes intensively managed to reduce potential spillover transmission. This is the case of the hazing of bison (*Bison bison*) around Yellowstone National Park due to brucellosis (White et al. 2011) or badger culling to prevent cattle tuberculosis (Donnelly et al. 2006). Underestimating CST can decrease the efficiency of measures aiming to stop disease spread by focusing only on within-species transmission (WST), while overestimating CST can lead to unnecessary measures aiming to stop CST when most disease transmission happens within a single species.

Several studies have focused on defining CST scenarios based on disease prevalence, e.g., ‘rare spillover events’ versus ‘multihost systems’ (Haydon et al. 2002; Dobson 2004; Fenton and Pedersen 2005). However, detecting CST and estimating its rate based only on prevalence data remains challenging. On the other hand, the explosive development of molecular techniques has opened new possibilities for using phylogenetic analysis of parasite genetics to infer epidemiological parameters (Grenfell et al. 2004; Archie et al. 2009; Didelot et al. 2012). Genetic techniques to study transmission were first used for fast evolving RNA viruses (Pybus and Rambaut 2009). In contrast, several bacterial pathogens harbor low DNA sequence diversity (Comas et al. 2009), limiting the inferences that could be made using genetic markers. Genetic studies of bacteria previously focused on variable number tandem repeat (VNTR) data (Lindstedt 2005) and, more recently, single nucleotide polymorphisms (SNPs) derived from whole-genome sequencing (Pearson et al. 2009; Didelot et al. 2012). The low cost and high mutation rates of VNTRs made them particularly useful to detect genetic differences in recent outbreaks (Lindstedt 2005). SNPs have a lower mutation rate per locus than VNTRs but deliver more stable and reliable genetic relationships between bacteria isolates, which is more suitable for studies on bacterial phylogenies (Foster et al. 2009). Both of these marker types have great potential and are now being used to answer a range of epidemiological questions, although reduction in cost of whole-genome sequencing will probably favor the use of SNPs rather than VNTRs in the near future (Achtman 2008).

Studies focusing on CST using VNTRs or SNPs have mainly described differences in bacteria genotypes between the two host species, and some have reconstructed the bacteria phylogeny using a clustering analysis, a phylogenetic tree or a network approach (see Table 1 for examples on identifying CST using genetic markers). However, these analyses have been conducted with relatively small sample sizes (especially in the wildlife species) and to our knowledge, no study has yet estimated CST rates using bacterial genetic markers (for viruses see Streicker et al. 2010). Therefore, it remains unknown whether the use of bacterial VNTRs and SNPs allows accurate estimation of CST rates, and what factors influence this estimation. Here, we used a simulation model where the true rates of transmission and mutation were known, to evaluate the ability of VNTRs and SNPs to correctly estimate rates of CST between two species (or populations).

**Table 1:** Example published studies focusing on CST between humans, livestock and wildlife using genetic markers.

Bacteria studies	Species involved and number of isolates (*n*)	Marker used	Method	Study Conclusion	References
Brucellosis at the Greater Yellowstone Ecosystem (GYE)	Cattle (23), elk (25), bison (10)	VNTR (10 loci)	Haplotype Network	CST from elk to cattle	Beja-Pereira et al. (2009)
Brucellosis at GYE	Cattle (43), elk (77), bison (196)	VNTR (10 loci)	Unweighted Pair Group Method with Arithmetic Mean (UPGMA) and Minimum Spanning Tree (MST)	CST from elk to cattle	Higgins et al. (2012)
Bovine Tuberculosis (TB) in Portugal	Cattle (157), wild boar (4), red deer (13), goat (7)	VNTR (8 loci)	UMPGA and MST	CST between cattle and wildlife	Duarte et al. (2010)
Bovine TB in Corsica	cattle (5), pig (2), wild boar (9)	VNTR (5 loci) combined with Spoligotype	Comparison of VNTR genotypes	CST between wild boar and cattle suggested	Richomme et al. (2010)
Bovine TB in Spain	Wild boar (21), red deer (10), fallow deer (14), I berian Lynx (4), fox (2), cattle (41)	VNTR (8 loci) combined with Spoligotype	Comparison of VNTR genotypes	CST between wildlife and cattle	Romero et al. (2008)
Bovine TB in Northern Ireland	Badgers (5), cattle (26)	38 SNPs from Whole-genome sequence	Comparison of SNPs	CST between badger and cattle	Biek et al. (2012)
Paratuberculosis in Germany	Cattle (40), red-deer (13)	VNTR (8 loci) combined with other markers (SSR and RLFP)	Comparison of VNTR genotypes	CST between cattle and deer suspected	Fritsch et al. (2012)
Paratuberculosis in Europe	Cattle (52), sheep (26),goat (32), several wildlife species (54)	VNTR (8 loci) combined with other markers (PFGE, AFLP, RFLP)	Comparison of VNTR genotypes	CST between wildlife and cattle	Stevenson et al. (2009)
Leprosy in the US	Armadillo (33), human (39)	51 SNPs from Whole-genome sequence combined with VNTR (10 loci)	MST on SNPs and VNTRs	Possible CST from Armadillos to humans	Truman et al. (2011)
Salmonella in the UK	Human (186), poultry (190), pigs (195)	VNTR (5 loci) combined with PFGE	Ward algorithm dendogram	Possible CST from domestic animals to humans	Best et al. (2007)
Escherichia coli O157:H7 in the US	Feral swine (13), cattle (26)	VNTR (10 loci)	Comparison of unique VNTR alleles and MST	CST between cattle and swine	Jay et al. (2007)

The clonal population structure of bacteria (Smith et al. 1993; Haubold et al. 1998) and other pathogens favors the use of a phylogenetic approach to infer bacterial migration patterns between hosts or locations (Selander et al. 1990; Spratt and Maiden 1999; Supply et al. 2003; Grenfell et al. 2004). Several well-studied methods in molecular phylogeny are available to reconstruct a parasite transmission history (Yang and Rannala 2012). Within this phylogenetic framework, host species identity can be considered as a character in the parasite phylogeny. Therefore, CST can be estimated as the number of character changes within the phylogeny using methods such as the most parsimonious reconstruction (MPR) (Slatkin and Maddison 1989; Cunningham et al. 1998) or more complex Bayesian inference approaches (Ronquist 2004; Lemey et al. 2009; Faria et al. 2013).

The most widely used MPR method assigns character states to interior nodes on the tree, minimizing the number of inferred changes in character state that are consistent with the observed data (Yang and Rannala 2012). This allows a rapid and intuitive reconstruction of ancestral states and provides a number of character changes within the phylogeny (Cunningham et al. 1998). When the ‘character’ under consideration is host species identity, the number of state changes provides an estimate of CST events. However, this method does not incorporate any mechanistic description of the process by which CST occurs and can be misleading when rates of evolution are fast or transmission to and from a particular species do not have the same probability (Cunningham et al. 1998; Yang and Rannala 2012). Alternatively, Bayesian inference of character evolution methods such as the character diffusion model (Ronquist 2004; Lemey et al. 2009) are currently being developed for the study of CST in RNA viruses such as rabies (Streicker et al. 2010; Faria et al. 2013) and account for tree uncertainty and more complex scenarios. However, they are more computationally intensive, making the evaluation of their performance (using numerous simulations) difficult. Bayesian methods also require knowledge to set prior values for parameters that are generally poorly known in bacterial systems (Yang and Rannala 2012). Here, we focus on testing the accuracy of CST estimations using the MPR method based on VNTR or SNP markers. We also tested the sensitivity of the estimates to several factors that will likely affect any phylogeny reconstruction, regardless of the method used.

We compared the ability of VNTRs and SNPs to reconstruct a known bacterial phylogeny and estimate CST rates by developing a discrete time susceptible-infectious-recovered individual-based stochastic model with two species (A and B). WST and CST rates were set to known constant values. For each stochastic simulation, we counted the number of both types of transmission and calculated *ϕ,* the percentage of all transmission that occurred across host species. In the model, we tracked the VNTR and SNP bacterial genotype of each infected host, with a defined mutational process for each genetic marker. At the end of each simulation, infected individuals from the population were sampled, and the phylogeny of the bacteria was reconstructed from the simulated genetic markers. From the phylogeny, we estimated 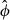 using a MPR algorithm (Narushima and Hanazawa 1997). We explored how bacterial phylogenetic reconstruction and our ability to estimate CST is affected by the following: (i) the number of mutations accumulated in the bacteria of each host species after bacteria introduction, (ii) the genetic similarity established before introduction between the strains introduced in both hosts, and (iii) the sample sizes of isolates within each host species. Finally, we discuss other factors influencing the reconstruction of phylogenies to reliably assess CST.

## Materials and methods

We simulated a scenario where the bacteria are introduced in both species A and B at the beginning of the simulation and then both WST and CST can occur. At the beginning of each simulation, one individual of each population was infected with a bacterial strain. Details on the transmission model are given in Appendix A.

### Two introduction scenarios

We explored two introduction scenarios. In the first scenario, both strains introduced at time zero in species A and B were identical in their VNTR or SNPs. This represents cases where both species are infected by the same strain from another species at roughly the same time. For example, brucellosis in bison (*Bison bison*) and elk (*Cervus canadensis*) in the Greater Yellowstone Ecosystem was introduced by European cattle (*Bos taurus*) at the beginning of the twentieth century (Cheville et al. 1998) (Table 1). This scenario is equivalent to having no bacteria genetic diversity generated in species A before the first CST event into species B, because the number of mutations accumulated prior to CST is low. In the second scenario, strains introduced in each species were genetically different. The difference between the introduced strains was five repeats at each VNTR locus or 50 SNPs. This scenario illustrates cases where strains in the two host species are already genetically different before CST occurs. This is a possible scenario for bacteria evolving in several species hundreds or thousands of years ago, with occasional CST between species. This may be the case for most gastrointestinal bacteria such as *E. coli* and probably the case for endemic bovine tuberculosis in wildlife reservoirs in Africa, Europe, and North America (Cosivi et al. 1998; Delahay et al. 2001; Aranaz et al. 2004; Wobeser 2009; Tenaillon et al. 2010). This is also equivalent to a scenario where introduction happens at the same time, but each species receives a different strain from a genetically diverse bacteria population in the contamination source. A third scenario where the bacteria evolve first in one species, and then CST occurs, is intermediate between the two extreme scenarios presented. Outcomes of this model should be (i) closer to the first scenario if genetic variability is low previous to the first CST scenario, or (ii) closer to the second scenario, if genetic variability of bacteria in species A before CST to species B is high. However, we did not test this scenario because it requires the addition of extra parameters to the model (e.g., time of evolution in one species before the first CST event and random selection of the strain transmitted from species A).

### Genetic markers

#### VNTR

Each infected individual contains a single pathogen strain characterized by several VNTR markers. Each VNTR locus consists of short nucleotide sequences that are repeated in tandem, and the number of repeats (considered as alleles) varies among genotypes (Vogler et al. 2006). We performed a limited review of 30 randomly selected studies that obtained bacterial VNTR genotypes and calculated an average of 10 [range from 4 to 49] VNTR loci used per study. Thus, we performed simulations for 10 loci (referred to as 10-VNTR) and the maximum value of 50 loci (referred to as 50-VNTR) (Le Flèche et al. 2001). For simplicity, all loci had the same mutation rate *θ*. We varied *θ* to produce different values of allelic variation (AV = average number of alleles per locus). Specifically, we chose to simulate AV = 2, 5, and 15, which correspond to low, medium, and high values of AV observed in different empirical systems (Keim et al. 2000; Farlow et al. 2002; Bricker and Ewalt 2005). Repeat copy number variation at these loci is the result of mutations resulting in the gain or loss of some number of repeats, known as the multistep mutation model (Fan and Chu 2007). This model is empirically supported as the mutation model for several bacteria (Vogler et al. 2006, 2007). If mutation occurred (at rate *θ*), the probability of mutating from x repeats to x ± *n* repeats was drawn from Vogler’s study on *Escherichia coli*, one of the few focusing on the mutation mechanisms of VNTR (Vogler et al. 2006). These probabilities were fixed to *P*(*n* = 1) = 0.75, *P*(*n* = 2) = 0.13, *P*(*n* = 3) = 0.04, *P*(*n* = 4) = 0.03, *P*(*n* = 5) = 0.02, and *P*(*n* = 6–10) = 0.03. Adding or subtracting a number of *n* repeats had equal probability (Vogler et al. 2006). A VNTR locus can mutate back to a previous number of repeats, which can generate genotypes that are identical, but not by descent. Detection of such cases, known as ‘homoplasy’ (Reyes et al. 2012), depends on the resolution of the genetic data and sampling. Homoplasy can cause erroneous inference about the genetic similarity between isolates and is especially problematic after many generations of isolation between lineages.

#### Single nucleotide polymorphism

Single nucleotide polymorphisms (SNPs) are single nucleotides in the bacterial genome that vary due to random point mutations, horizontal gene transfer or intragenic recombination (Brumfield et al. 2003; Pearson et al. 2009). SNPs can theoretically occur at any nucleotide throughout a genome and because nucleotides have relatively low mutation rates compared with VNTRs, multiple mutations at a single site are unlikely (Brumfield et al. 2003). Thus, most SNPs are only bi-allelic (i.e., only two nucleotide states are observed) and are typically not affected by homoplasy (Pearson et al. 2009). The declining cost of DNA sequencing (SNPs are identified by flanking sequences) should facilitate the discovery and genotyping of SNPs in many bacterial genomes, thus likely increasing their use as bacterial genetic markers in the near future (Achtman 2008). In this model, we mimic a set of SNPs by a string of binary integers (0 or 1). At each time step, each nucleotide can mutate with probability *ω*. We only allowed each nucleotide to mutate once. To reduce computational time, the bacterial genome was simulated by a 10 000 nucleotide string. Different mutation rates allowed an accumulation of 100–1000 variable SNPs after introduction. Although up to 10 000 SNPs have been identified for *Mycobacterium tuberculosis* worldwide (Achtman 2012), many bacteria show less than a hundred informative SNPs at the geographic scale relevant to epidemiological studies, for example, around 100 for brucellosis at the Greater Yellowstone Ecosystem (Foster et al. 2009), 38 for *M. bovis* strains in Northern Ireland that have identical VNTR genotype (Biek et al. 2012), and 51 for *M. leprae* in the United States (Truman et al. 2011).

### Phylogenetic reconstruction and CST estimation

We reconstructed phylogenies from both VNTR and SNPs using a neighbor-joining (NJ) tree method (Saitou and Nei 1987), from a pairwise matrix of genetic distance between strains. The NJ method is widely used to reconstruct bacteria phylogenies using both of these markers (Klevytska et al. 2001; Chen et al. 2007; Comas et al. 2009; Monot et al. 2009). The MPR algorithm (Narushima and Hanazawa 1997) was used to estimate *c*, the minimum number of character changes necessary to construct a tree compatible with the matrix. Although *c* is considered to be proportional to the number of CST events, no analytical relationship has been established to estimate CST from *c* (Slatkin and Maddison 1989). We suggest that the true percentage of CST 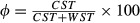 is approximated by the estimated percentage 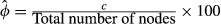. Phylogenetic analyses were performed using the ape package in R 2.15.2 (R Development Core Team 2012). Model initialization and parameter values are detailed in Appendix A.

## Results

**Figure 1:**
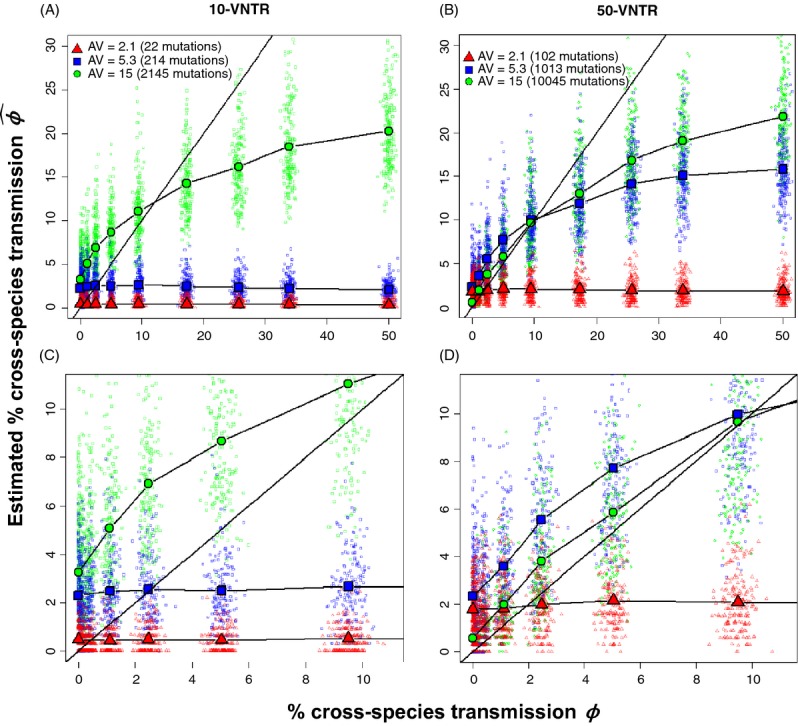
Relationship between true and estimated percentage of cross-species transmission using VNTRs when the same strain is introduced. The simulated percentage of CST, *ϕ*, compared with its estimation, 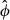, using the MPR algorithm in a scenario where the strains introduced in each species were identical. Colored points represent each of the 200 simulations per value of *β*, whereas each line illustrates the average relationship between the realized and estimated value (points averaged over the same value of *β*). The straight line represents a theoretical un-biased estimation. In (A) 10 loci were used, with the average number of total mutations accumulated since introduction equal to 22, 214 and 2145. In (B) 50 loci were used, with the average number of total mutations accumulated equal to 102, 1013 and 10045. A zoomed plot of 0-10% CST is shown for (A) and B in (C) and (D), respectively.

**Figure 2:**
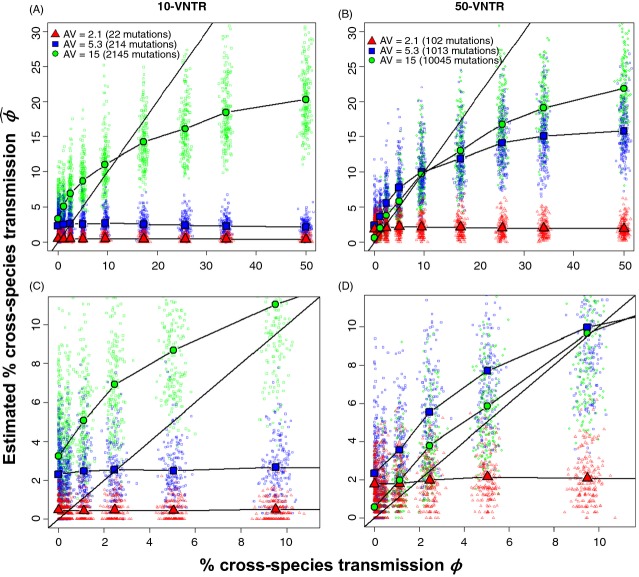
Relationship between true and estimated percentage of cross-species transmission using VNTRs when different strains are introduced. The simulated percentage of CST, *ϕ*, compared with its estimation, 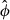, using the MPR algorithm in a scenario where the strains introduced in each species were different at each loci by five repeats. Colored points represent each of the 200 simulations per value of *β*, whereas each line illustrates the average relationship between the realized and estimated value (points averaged over the same value of *β*). The straight line represents a theoretical un-biased estimation. In (A) 10 loci were used, with the average number of total mutations accumulated since introduction equal to 22, 214, and 2145. In (B), 50 loci used with the average number of total mutations accumulated equal to 102, 1013, and 10045. A zoomed plot of 0–10% CST is shown for (A) and (B) in (C) and (D), respectively.

**Figure 3:**
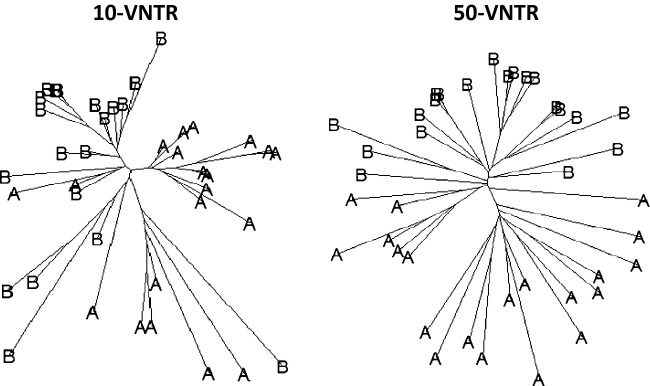
Phylogenetic reconstructions of a representative scenario with no CST transmission using 10 and 50 VNTRs. A NJ tree was reconstructed for 20 randomly selected infected individuals using either 10-VNTR or 50-VNTR with the same individuals sampled in both cases. In this scenario, there was no cross-species transmission, AV = 15.1, and the same strain was introduced in both species.

At a low number of mutations (allelic variation AV ≈ 2), the estimated percentage of CST, 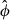, was uncorrelated with the actual percentage of CST in the simulation, *ϕ*, for both 10 and 50-VNTR (Figs 1 and 2). *ϕ* and 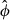 were more correlated when *ϕ* was less than 10%, allelic variation was high, and more VNTRs were used. In all cases, the estimated 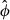
s from each simulation were highly variable (Figs 1 and 2). 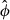 was an underestimate of *ϕ* whenever CST was frequent. When the same strain is introduced in both species and *ϕ* = 0, a medium or high number of mutations could produce 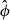 ranging from 0 to 9% when using 10 VNTRs (Fig. 1C). This shows that high mutation rates can generate false detections of CST. This was less common using 50-VNTR. This phenomenon can be visualized in Fig. 3, where reconstructing the phylogeny using 10-VNTR with *ϕ* = 0 falsely concluded that CST happened on several occasions, while the same phylogeny using 50-VNTR showed no evidence of CST. Overall, the MPR method tended to underestimate *ϕ* when its value exceeded 10%.

**Figure 4:**
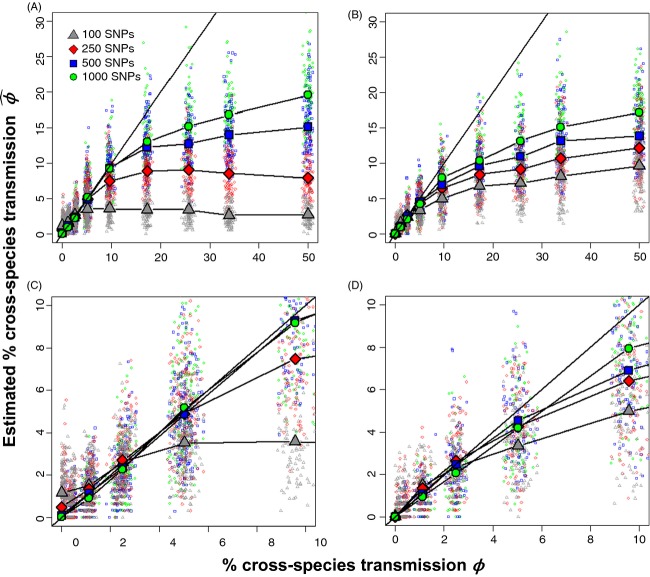
Relationship between true and estimates of the percentage of cross-species transmission using SNPs. The simulated percentage of CST, *ϕ*, compared with its estimation, 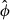, using the MPR algorithm. Colored points represent each simulation per value of *β,* whereas each line illustrates the average relationship between the realized and estimated value (points averaged over the same value of *β*). Different lines show different numbers of informative SNPs (going from 100 to 1000). The straight line represents a theoretical un-biased estimation. In (A), the same strain was introduced. In (B), strains introduced in each species differed by 50 SNPs. A zoomed plot of 0–10% CST is shown for (A) and (B) in (C) and (D), respectively.

Estimations of *ϕ* using SNPs were usually less biased than those using VNTR, especially when *ϕ* < 5%, and this estimate is improved by increasing the number of SNPs (Fig. 4). However, 100 SNPs still resulted in highly biased estimates of CST, in a scenario where the same strain was introduced in both species (Fig. 4A, C). Values of 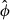 using 250 SNPs were within 20% bias of the actual value when *ϕ* < 5%. Values of 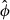 using 500 and 1000 SNPs were unbiased when *ϕ* < 10%, although stochastic variation could generate simulations over (or under) *ϕ* by up to 100% (Fig. 4A, C). Similar to the VNTR results, 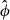 was biased low when CST was frequent. Values of 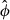 were less biased for all number of SNPs when the introduced strains were genetically different and *ϕ* < 3%. However, this initial difference in strains also generated a more pronounced underestimation for *ϕ* > 3% (Fig. 4D).

Introducing genetically different strains to the two hosts allowed a better estimation of *ϕ* using VNTR data when *ϕ* < 10% and in SNPs when *ϕ* < 3%. Even with 500–1000 SNPs and different host strains, we underestimated the percentage of CST when *ϕ* was between 5 and 10 percent. When *ϕ* > 10%, relatively small differences between introduction scenarios were observed, and general underestimation was mostly a consequence of using the MPR method.

**Figure 5:**
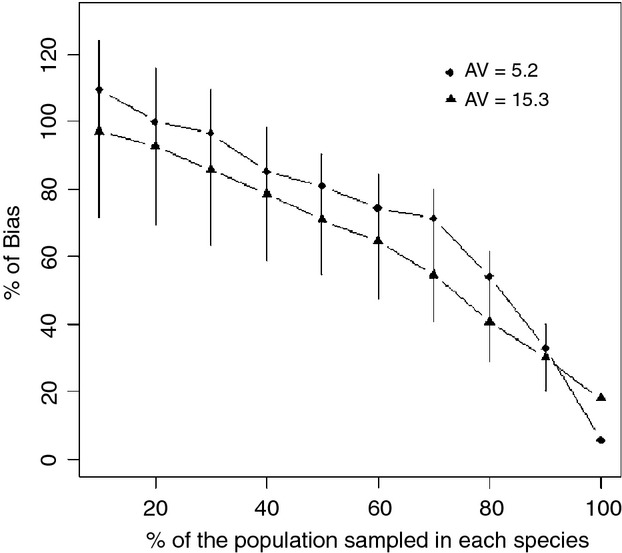
The influence of sample size on 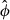. The percent 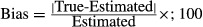 in 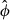 decreased as the sampling percentage of the infected populations approached 100%. For this simulation, we assumed that *ϕ* = 10%, 50-VNTR, and an allelic variation (AV) equal to 5.2 or 15.3. Each point is an average of 400 random samplings for a given simulation and sampling intensity. Error bars represent standard errors of the mean.

**Figure 6:**
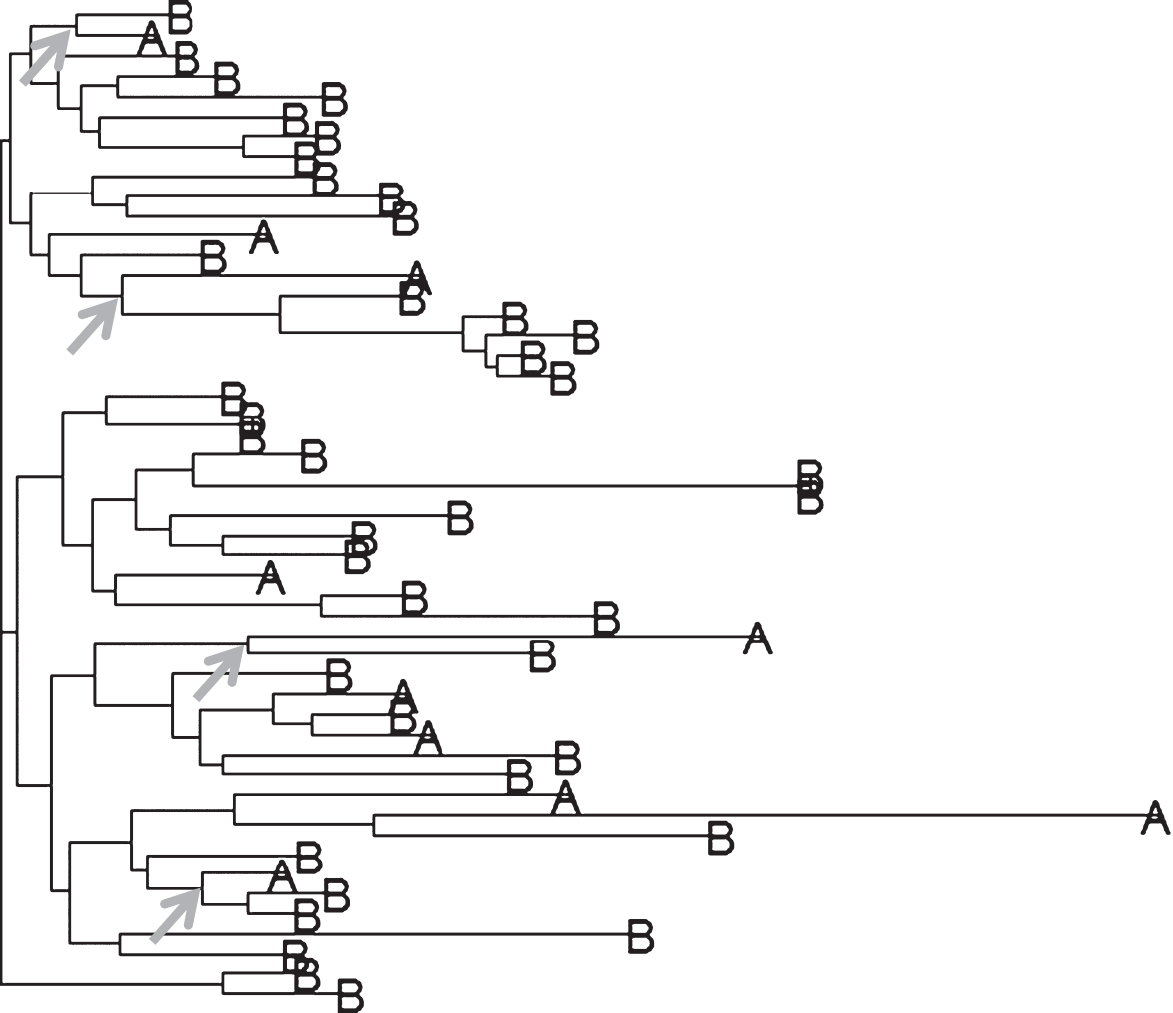
Phylogenetic reconstructions with unbalanced sampling in a scenario of transmission only from A to B. A NJ tree was reconstructed with a sample size of 10 individuals for species A and 40 for species B. Phylogenetic reconstruction from a randomly selected run from a scenario where CST only happens from A to B. Based on a visual assessment, species B seems to be transmitting the bacteria to species A (indicated by some of the gray arrows), which does not occur in this model. Parameter values: *ϕ =* 33%, 10-VNTR and AV = 5.9. The tree was rooted to infer directionality. Similar results were obtained using 50-VNTR or 1000 SNPs.

**Figure B1:**
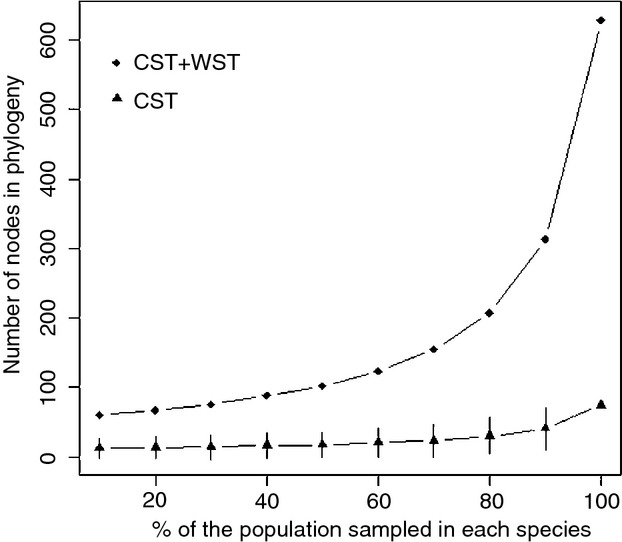
The influence of sample size on CST estimation. The number of nodes identified as CST in the phylogeny (CST line) and the total number of nodes (CST+WST line) are estimated for the same simulation run as the one used in Figure 5. These numbers are presented as a function of the percentage of population sampled.

Lower proportions of infected individuals sampled resulted in larger overestimates of *ϕ* (Fig. 5). Our results were similar regardless of whether we used 10, 50-VNTR, or 1000 SNPs. The number of CST identified in the phylogeny increased with the percentage of individuals sampled (Fig. B1). However, the total number of events (nodes) detected in the phylogeny (WST + CST) also increased but with a bigger slope than for CST events (Fig. B1). This generated a higher bias of 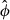 for low sample sizes. For example, sampling 10% of the population doubled the estimated 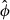 compared with sampling the entire population (Fig. 5). An unbalanced sample size of 10 and 40 for species A and B, respectively, may mislead a researcher to conclude that species B is transmitting bacteria to species A in a scenario where transmission only occurred from A to B (Fig. 6).

## Discussion

Estimates of bacterial CST based on the most parsimonious phylogeny reconstructed using VNTR markers tend to be biased across a wide range of the parameter space we explored. Less biased and variable estimates of CST are possible using a large number of SNPs and when the percentage of all transmission that is across species is less than 10. In general, CST rate estimates were most reliable in systems with more mutations, markers, and high genetic differences between introduced strains. Subsampling the infected population tended to result in overestimates of CST. The effect of stochasticity was also substantial using both SNPs and VNTRs, suggesting that estimations of CST rates will be generated with large uncertainty over the precise value. Although we focused on bacteria, the above factors would play a similar role for other clonal pathogens.

In general, bias in the estimation of CST rates using VNTRs can be attributed to a poor reconstruction of the bacteria phylogeny with some ancestor nodes being wrongly assigned. On the one hand, overestimation of low CST rates can be exacerbated by the effects of homoplasy when the number of markers is small and mutation rate is high. On the other hand, underestimation of high CST rates can be attributed to the parsimonious nature of this MPR algorithm, which minimizes the number of CST necessary to reconstruct the phylogeny. The latter problem also occurs when using SNPs. Little is known about the mutation rate of VNTRs in most bacterial species (Vogler et al. 2007). Therefore, if these markers are used to estimate CST rates given their simple and cost-effective implementation, our results suggest that their mutation rate needs to be estimated, that more than 50 markers are necessary and that allelic variability per marker should be high. This requires evaluating whether identifying this amount of variable markers is achievable and economically viable compared with other methods such as SNPs.

Single nucleotide polymorphisms present the advantage that estimations of their substitution rate per genome are now becoming more available for bacteria (Achtman 2012). Our results show that estimations of CST rates lower than 5% can be achieved with relatively low bias using as low as 250 SNPs. This confirms empirical results suggesting that the stability of SNPs is more useful to disentangle bacteria evolutionary history compared to VNTRs (Comas et al. 2009). For values higher than 10%, the MPR method tends to underestimate CST and does not seem suited for this purpose. Instead, other methods such as Likelihood or Bayesian analysis of character changes within a phylogeny might be more accurate (Ronquist 2004), although their efficiency also needs to be tested in a simulation framework. No estimation of bacterial CST rates has been achieved so far for empirical systems so it is hard to determine in advance the possible range of *ϕ* for a particular CST empirical system (but see Streicker et al. 2010 in bat rabies for an estimation of similar parameters). However, we expect that individuals interact predominantly with members of their own species and thus *ϕ <* 10% in most systems, encouraging the use of SNPs when studying CST. However, until estimates are available, advancing a CST rate for a given system is mostly arbitrary, unless prevalence data on both species can help inferring epidemiological parameters.

Contrary to VNTRs, phylogenies using SNPs are more stable and homoplasy is reduced. However, if the number of informative SNPs and the CST rates are low, the lack of genetic differentiation between bacteria from different host species can still lead to an overestimation of CST rates because similarities in strains derived from a common introduction will be wrongly attributed to CST events. Given the relatively low mutation rate of SNPs, even 250 SNPs can be difficult to accumulate in systems where bacteria introduction is relatively recent (see examples given in the methods section). Thus, our results encourage the current effort to increase the number of informative SNPs available for bacterial pathogens using comparative genomics (Pearson et al. 2009; Achtman 2012). Most viruses have higher substitution rates than bacteria. Thus, the number of markers necessary to achieve a reliable estimation of viral CST should be easier to obtain.

Most bacteria populations will experience population bottlenecks when introduced into a new geographic area or jumping to a different host species (Smith et al. 2006; Achtman 2008). Our two extreme scenarios of bacteria introduction (identical or different genetic strains introduced within each species), provided insights into the importance of initial bottlenecks when estimating CST rates. Overall, our results suggest that initial genetic differences between strains introduced into the system can either increase or decrease the precision in the estimates of CST, depending on the number of SNPs used and the actual value of the CST rate. In most systems, assessing genetic differences between strains at the time of introduction (or host species jump) can be challenging and requires a previous estimation of both mutation rate and time since introduction. However, this knowledge is necessary to disentangle genetic differences in strains between host species that are due to new accumulated mutations since introduction, or to differences existing prior to bacteria introduction. New approaches applying Bayesian statistics to genetic data have shown promising results elucidating this type of problem (Sousa et al. 2012).

Methodological issues can also affect the estimation of CST rates from genetic data. In most empirical systems, especially focusing on wildlife (Biek et al. 2012; Richomme et al. 2012), only a very small percentage of the host (and bacteria) population is sampled. It was expected that the estimated 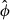 would increase with sample size, since genotypic diversity almost universally increases with it (Wolda 1981). Our results, however, showed the opposite pattern. CST was overestimated when sampling a small percentage of the population. In the case presented here, the estimated percentage of CST was about two times higher than what was simulated when sampling 10% of the population. The total size of the infected population (around 500 individuals in our simulations) and the sample size used to reconstruct the phylogeny will also influence the amount of variation around the estimates generated by stochasticity. Another recurrent sampling problem in empirical systems is that sampling is almost always unbalanced among host species (Biek et al. 2012; Higgins et al. 2012). Our results suggest that unbalanced sampling can substantially affect the phylogenetic reconstruction and conclusions inferred from that phylogeny. In our simple scenario, we showed how CST direction can be wrongly interpreted from a phylogeny using unbalanced sampling. Sampling a large portion of each species is almost never achieved (see Table 1), especially when working with wildlife species. Therefore, using a simulation approach to study their impact on CST rates such as the one presented here might be an alternative way to correct for bias related to sampling effort.

### Other factors influencing phylogeny reconstruction and CST estimation

Several assumptions of our model are simplistic representations of reality and understanding how their relaxation would influence estimates of CST rates requires further investigation. For example, CST was modeled as a constant rate per time step, but CST events could be clustered in time (e.g., only in years with particular environmental conditions). This will generate a more heterogeneous phylogeny than the ones analyzed in this model, which could influence the estimations of CST rates. Furthermore, all individuals were simultaneously sampled at the end of a simulation, but several data sets of bacteria include samples that have been collected over the course of an outbreak. Samples collected at a similar time could cluster together in the phylogeny and affect the estimations of CST. In this model, we also fixed the time of bacteria introduction and we introduced only one strain in each species. However, the time of bacteria introduction in many empirical systems remains unknown. The MPR method used here does not include information about time (e.g., branch length) and is therefore not suited to infer parameters such as the time of first introduction. Finally, we used a simple model of mutation rate, particularly for VNTR, where all loci had the same mutation rate. Understanding consequences of applying more complex and realistic models of mutation, for example, with different sections of the genome mutating at different rates (Barrick et al. 2009), will require further work.

There are several other methodological and epidemiological factors influencing estimates of CST rates that we do not explore in this model. Methodological factors include for example that (i) different clustering methods such as the NJ tree, minimum spanning tree (Teh et al. 2010), UPGMA (Davis et al. 2009) do not generate the same phylogenetic reconstruction (results obtained from simulations, data not shown) but are all used in different studies focusing on VNTR, (ii) SNP discovery bias reduces the amount of informative SNPs available (Pearson et al. 2009), and (iii) host spatial clustering can also generate phylogenetic clustering (Ruzzante et al. 1996). Other factors related to bacteria evolution making CST rates difficult to estimate include (i) host immune system selection of particular strains in different species (Brunham et al. 1993), (ii) bacteria recombination affecting phylogenetic reconstruction (Feil et al. 2001), (iii) within-host evolution of the bacteria (Gyuranecz et al. 2013), or (iv) changes in bacteria population through time (for viruses, see: Volz et al. 2009; Frost and Volz 2010). All or some of these factors may apply to a given system studied and should also be taken into account when trying to estimate CST rates from phylogenetic data.

Given the highlighted limitations in this study and the numerous factors influencing CST rates, we recommend that future studies pay particular attention to two main steps in the process of using genetic markers to estimate CST rates. First, the phylogenic tree used in the analysis needs to capture the underlying epidemiological process generating the tree. This will require a balanced sample between species and also an understanding of how much genetic variability of the bacteria is represented by the given sampling effort. Secondly, the strength of the analysis will depend on the amount of mutation accumulated since the pathogen was introduced in the system. Therefore, it is inevitable to focus efforts on estimating either time since introduction (e.g., from historical records of disease prevalence) or the substitution rate of the marker (e.g., from laboratory experiments or genomic comparative analysis). This is important not only when using the MPR method but also for Bayesian or Likelihood approaches that require an estimation of the mutation rate. Finally and sometimes forgotten, CST will also affect disease prevalence in the potential host. Thus, combining both epidemiological time series data with genetic data may be a powerful approach.

## Appendix A

### Transmission model

We used a stochastic discrete time model to simulate the disease dynamics in the two host species (A and B) assuming that each individual can move through three different classes: susceptible, infectious, and recovered (Keeling and Rohani 2008). Susceptible individuals of species *i* can be infected by infectious individuals of their species (I_*i*_) or infectious individuals of the alternative species (I_*j*_) with probability *p*_*i,t*_ per time step *t*, where *i* equals 1 or 2 and *i* ≠ *j*. Let *α*_*i*_ and *β*_*j*_ represent the probability of infection imposed by one infected individual either within-species or between-species, respectively. Using a Reed-Frost model of transmission, the probability that an individual of species *i* is infected in time step *t* is: . We present results from scenarios where the transmission rate within the species is the same (*α*_*i*_ = *α*_*j*_), and the CST is also the same (*β*_*i*_ = *β*_*j*_) but WST is more likely than between species (*α*_*i*_ > *β*_*i*_). Similar results are obtained when CST occurred in only one direction (*β*_*j*_ = 0).

When a transmission event occurs, one infected individual from either host species is randomly assigned to transmit its bacteria, and genetic markers, to the newly infected individual. The probability of assigning a bacterial genotype from its own species is given by . Each infected individual passes from the infectious to recovered state with probability *γ*. Following disease transmission, mortality and reproduction take place as a single death/birth pulse at the end of the year, keeping a constant population of size *N* = 1000 individuals in each species. Each individual dies and is replaced by a new susceptible individual with probability *μ*. There was no disease-induced mortality or population structure in this model.

### Model initialization and parameter values

After introduction, the model was run for 100 time steps. This time step can be considered as a host epidemiological time step, corresponding to the expected interval between bacterial transmission events. This time step coincided with the host generation time, during which the mortality/birth process takes place. The duration of the simulation was fixed, so the number of mutations accumulated since introduction only varied with the mutation rate parameter *θ* or *ω*. At the end of each simulation (*t* = 100), all infected individuals from both species were sampled to reconstruct the bacteria phylogeny and estimate *φ* unless stated otherwise. Although achieving 100% sampling coverage is unrealistic for most empirical systems, this scenario was used in order to study the effects of other parameters such as the number of mutations accumulated and strain introduction. Subsequently, we studied the effect of randomly selecting a proportion of individuals from the total population size. Several parameters were fixed in the model because we focused on exploring only the influence of mutation rate, genetic similarity of the introduced strain and sampling effort. We assumed that *u* = 0.06, *γ* = 0.05, *N* = 1000 individuals and α = 0.003 in both species. This allowed a disease prevalence of up to 50% in both species over 100 time steps. Changing the value of *α* in one or both species did not affect qualitatively the results presented. The probability of infection imposed by each infected individual of another species to a susceptible one, *β*, varied from 0 to 0.003 going from no CST to a scenario where CST = WST. All simulations were coded and run using Delphi v6 computing software (2006, Borland, Inc.). The code is available upon request to the corresponding author.
